# Supplementary material for: Association of MGMT Promoter and Enhancer Methylation with Genetic Variants, Clinical Parameters, and Demographic Characteristics in Glioblastoma
Source: Cancers (Basel). 2023 Dec 9;15(24):5777. doi: 10.3390/cancers15245777 (PMC10742072; doi:10.3390/cancers15245777)
Supplement: Supplementary file 1 [file cancers-15-05777-s001.zip › Supplementary_Figures.pdf]

Supplementary Figures

Association of *MGMT* Promoter and Enhancer Methylation with Genetic Variants, Clinical Parameters, and Demographic Characteristics in Glioblastoma

Katja Zappe [1], Katharina Pühringer [1], Simon Pflug [1] Daniel Berger [1], Serge Weis [2], Sabine Spiegl-Kreinecker [3], and Margit Cichna-Markl [1,*]

[1] Department of Analytical Chemistry, Faculty of Chemistry, University of Vienna, 1090 Vienna, Austria; katja.zappe@univie.ac.at (K.Z.); katharina.puehringer@univie.ac.at (K.P.); n12030136@students.meduniwien.ac.at (S.P.); office@berger-daniel.at (D.B.)

[2] Division of Neuropathology, Department of Pathology and Molecular Pathology, Kepler University Hospital GmbH, Johannes Kepler University, 4040 Linz, Austria; serge.weis@kepleruniklinikum.at

[3] Department of Neurosurgery, Kepler University Hospital GmbH, Johannes Kepler University, 4040 Linz, Austria; sabine.spiegl-kreinecker@kepleruniklinikum.at

* Correspondence: margit.cichna@univie.ac.at (MCM)

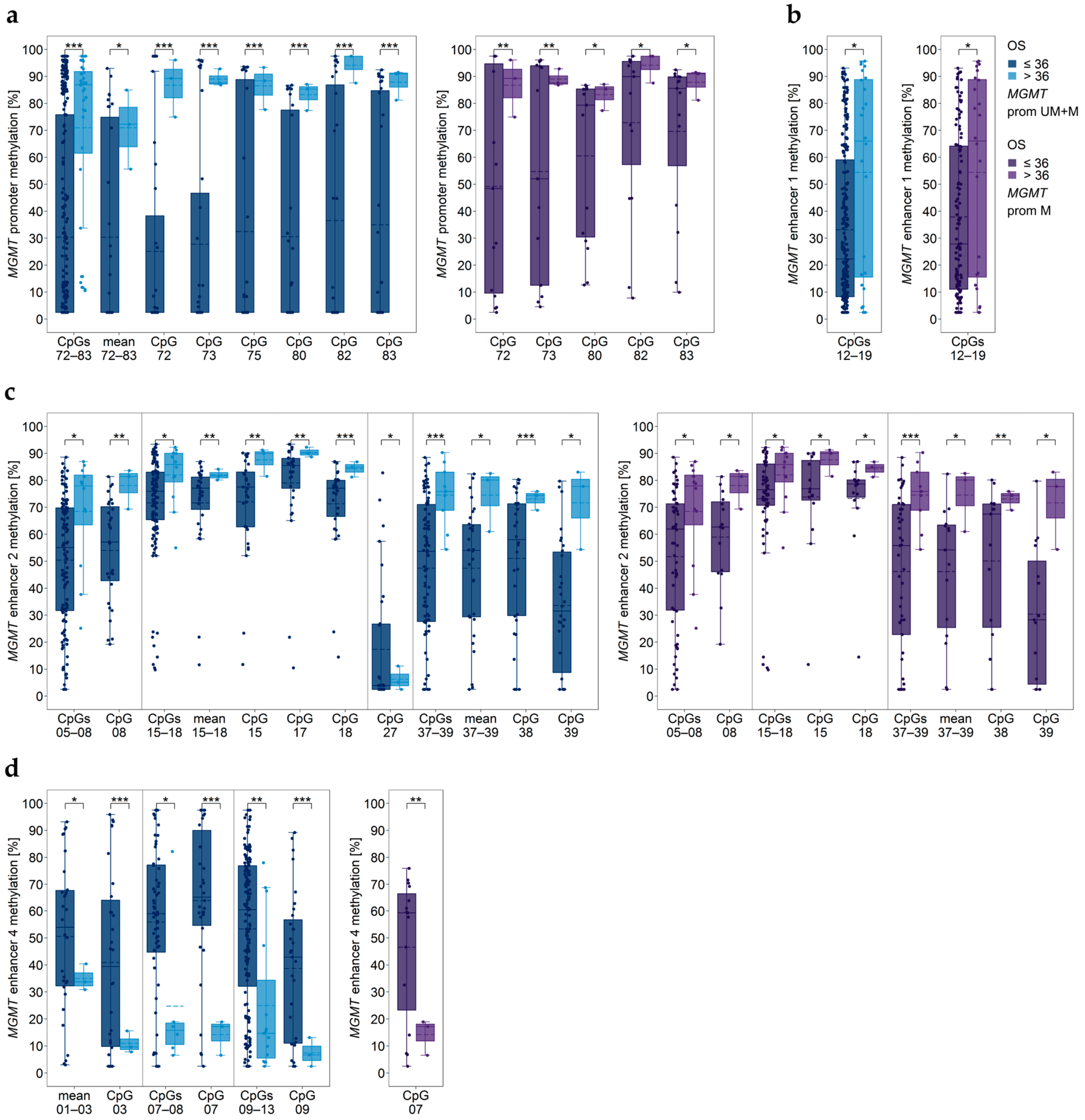

Figure S1. Significantly different *MGMT* promoter/enhancer methylation levels between non-long-term survivors (≤ 36 months) and long-term survivors (> 36 months). *MGMT* (**a**) promoter, (**b**) enhancer 1, (**c**) enhancer 2, and (**d**) enhancer 4 regions. prom M: promoter methylated (purple), UM: unmethylated, UM+M (blue) samples. Gray vertical lines separate data obtained with different assays. Significance levels: *p ≤ 0.05, **p ≤ 0.01, ***p ≤ 0.001. Data points represent the mean of two independent runs.

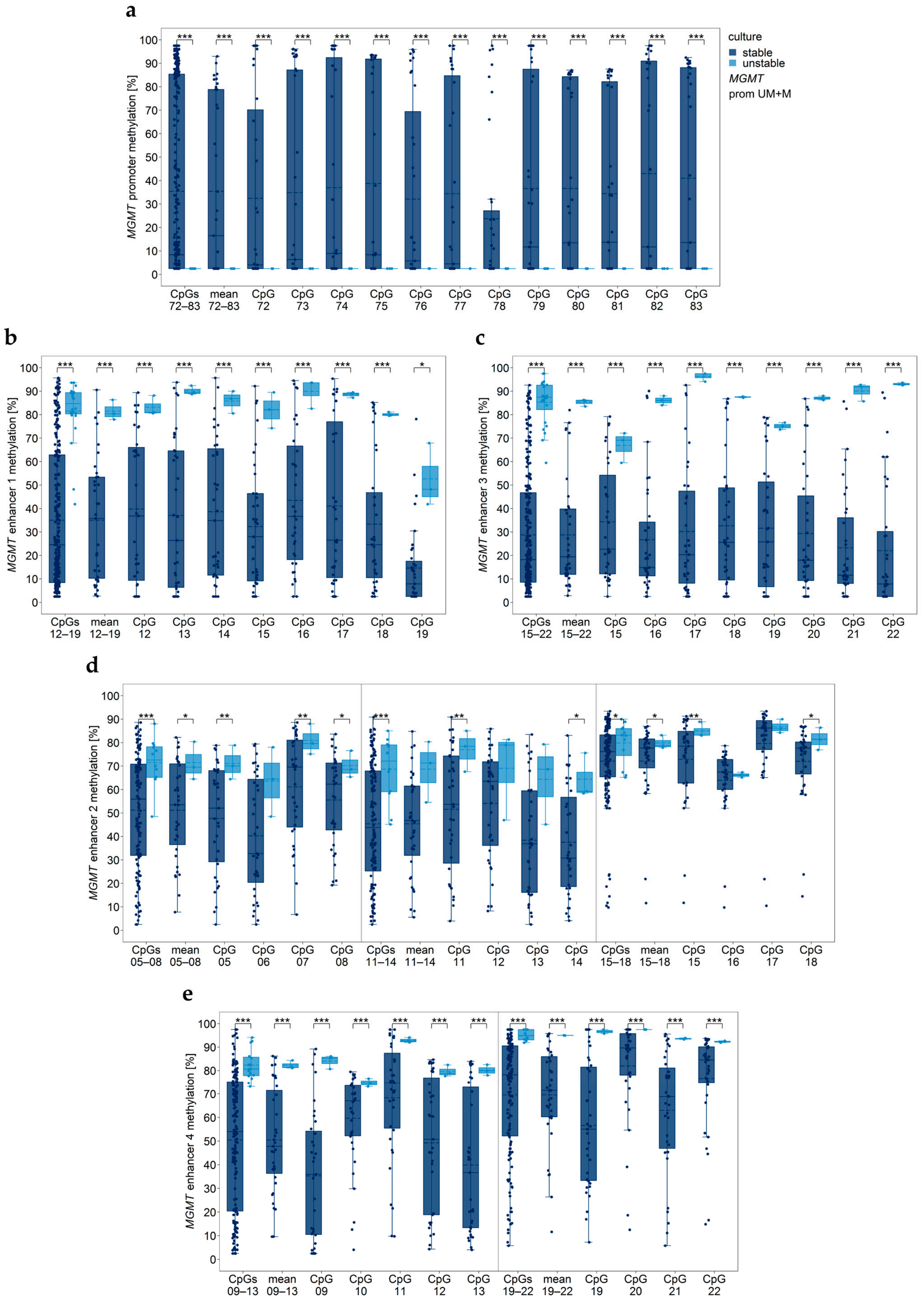

Figure S2. *MGMT* promoter/enhancer methylation levels of stable and unstable cell cultures. *MGMT* (**a**) promoter, (**b**) enhancer 1, (**c**) enhancer 3, (**d**) enhancer 2, and (**e**) enhancer 4 regions. Gray vertical lines separate data obtained with different assays. Significance levels: *p ≤ 0.05, **p ≤ 0.01, ***p ≤ 0.001. Data points represent the mean of two independent runs. Due to limited sample amount, GBM24, GBM36, and GBM37 were omitted from analysis of enhancer 2 CpGs 24–27 and CpGs 37–39 as well as enhancer 4 CpGs 01–03, CpGs 07–08. GBM36 was also not analyzed for enhancer 4 CpG 19–22. prom M: promoter methylated, UM: unmethylated.

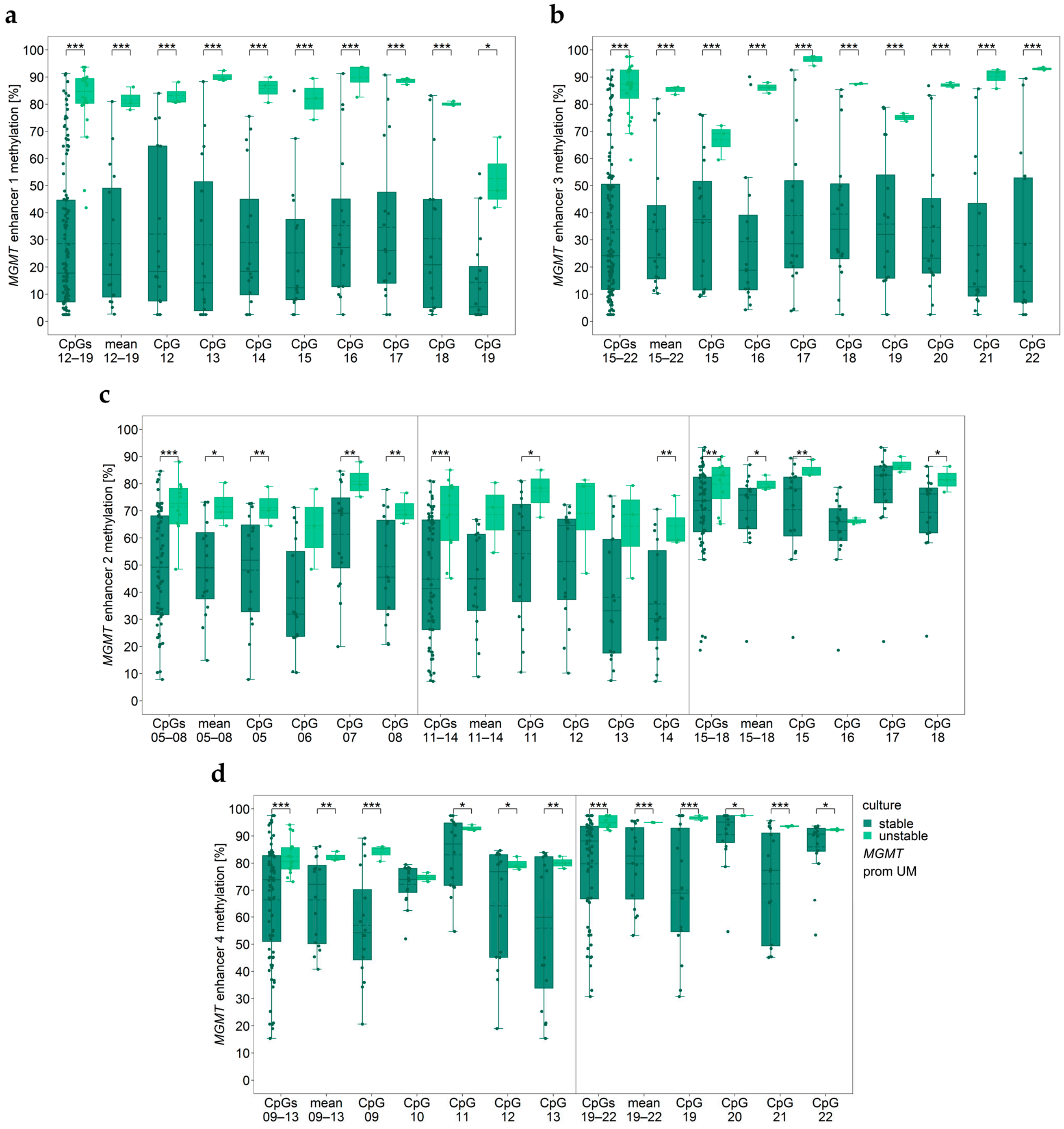

Figure S3. *MGMT* promoter/enhancer methylation levels of stable and unstable cell cultures in *MGMT* promoter unmethylated samples. *MGMT* (**a**) enhancer 1, (**b**) enhancer 3, (**c**) enhancer 2, and (**d**) enhancer 4 regions of promoter unmethylated patients. Gray vertical lines separate data obtained with different assays. Significance levels: $^{*}p \leq 0.05$, $^{**}p \leq 0.01$, $^{***}p \leq 0.001$. Data points represent the mean of two independent runs. Due to limited sample amount, GBM24, GBM36, and GBM37 were omitted from analysis of enhancer 2 CpGs 24–27 and CpGs 37–39 as well as enhancer 4 CpGs 01–03, CpGs 07–08. GBM36 was also not analyzed for enhancer 4 CpG 19–22. prom UM: promoter unmethylated.
