# Supplementary material for: Association of MGMT Promoter and Enhancer Methylation with Genetic Variants, Clinical Parameters, and Demographic Characteristics in Glioblastoma
Source: Cancers (Basel). 2023 Dec 9;15(24):5777. doi: 10.3390/cancers15245777 (PMC10742072; doi:10.3390/cancers15245777)
Supplement: Supplementary file 1 [file cancers-15-05777-s001.zip › Supplementary_Tables.pdf]

Supplementary Tables

Association of *MGMT* Promoter and Enhancer Methylation with Genetic Variants, Clinical Parameters, and Demographic Characteristics in Glioblastoma

Katja Zappe [1], Katharina Pühringer [1], Simon Pflug [1], Daniel Berger [1], Serge Weis [2], Sabine Spiegl-Kreinecker [3], and Margit Cichna-Markl [1,*]

[1] Department of Analytical Chemistry, Faculty of Chemistry, University of Vienna, 1090 Vienna, Austria; katja.zappe@univie.ac.at (K.Z.); katharina.puehringer@univie.ac.at (K.P.); n12030136@students.meduniwien.ac.at (S.P.); office@berger-daniel.at (D.B.)
[2] Division of Neuropathology, Department of Pathology and Molecular Pathology, Kepler University Hospital GmbH, Johannes Kepler University, 4040 Linz, Austria; serge.weis@kepleruniklinikum.at
[3] Department of Neurosurgery, Kepler University Hospital GmbH, Johannes Kepler University, 4040 Linz, Austria; sabine.spiegl-kreinecker@kepleruniklinikum.at
* Correspondence: margit.cichna@univie.ac.at (MCM)

Table S1. Clinical and demographic data of GBM patients for which stable cell cultures could not be established.

patient	age [y]	sex	KPS [%]	Ki-67 [%]	primary therapy	adjuvant therapy	OS [m]	PFS [m]	MGMT exp	*MGMT* rs16906252	*TERT* prom	*TERT* rs2853669
GBM36	62	m	50	≤ 50 (25)	none	none	1.48	n.s.	0.90	CT	wt	TT
GBM37	63	m	80	> 50 (60)	R-Ch-T	ABT-414	19.50	9.01	0.11	CT	wt	TT
GBM38	76	m	30	≤ 50 (20)	none	none	4.44	n.s.	0.29	CC	wt	CT

GBM36–38: glioblastoma multiforme patients. ABT-414: depatuxizumab mafodotin, Ch-T: chemotherapy, exp: expression, f: female, KPS: Karnofsky Performance Scale, m: male, mut: mutation, n.s.: not specified, OS: overall survival, PFS: progression-free survival, R-Ch-T: radio-chemotherapy, wt: wildtype.

Table S2. Interaction effects found by two-way ANOVA for significantly different (one-way ANOVA) *MGMT* promoter/enhancer methylation levels between *TERT* promoter wildtype and mutations, between *TERT* rs2853669 genotypes, and between patients with low (≤50%) and high (> 50%) Ki-67 index.

region	CpG	promoter	interaction	F-value	*p*-value
promoter	72–83	M	*TERT* prom * age	3.552	**0.030**
enhancer 2	37–39	UM+M	*TERT* prom * sex	10.034	**0.002**
		M	*TERT* prom * sex	4.416	**0.040**
	37	M	*TERT* prom * sex	5.812	**0.030**
enhancer 3	15–22	UM	*TERT* prom * sex	4.224	**0.042**
		M	*TERT* prom * age	37.604	**< 0.001**
			TERT prom * sex	29.500	**< 0.001**
promoter	72–83	UM+M	*TERT* rs2853669 * sex	27.778	**< 0.001**
enhancer 1	12–19	UM+M	*TERT* rs2853669 * sex	9.562	**0.002**
		UM	*TERT* rs2853669 * sex	4.272	**0.041**
		M	*TERT* rs2853669 * sex	7.793	**0.006**
enhancer 3	15–22	UM	*TERT* rs2853669 * sex	6.032	**0.015**
enhancer 4	19–22	UM	*TERT* rs2853669 * age	7.312	**0.009**
enhancer 1	12–19	UM+M	Ki-67 * sex	6.315	**0.013**
enhancer 2	05–08	UM+M	Ki-67 * age	8.103	**0.005**
			Ki-67 * sex	11.490	**0.001**

region	CpG	promoter	interaction	F-value	*p*-value
	08	UM+M	Ki-67 * sex	4.964	**0.038**
	37–39	UM+M	Ki-67 * age	7.081	**0.010**
			Ki-67 * sex	21.309	**< 0.001**
enhancer 3	15–22	M	Ki-67 * sex	9.233	**0.003**

M: methylated, prom: promoter, UM: unmethylated.

Table S3. Non-significant main effects identified by two-way ANOVA for significantly different (one-way ANOVA) *MGMT* promoter/enhancer methylation levels between *TERT* promoter wildtype and mutations, between *TERT* rs2853669 genotypes, and between patients with low (≤50%) and high (> 50%) Ki-67 index.

region	CpG	promoter	test	effect	one-way *p*-value	F-value	*p*-value
enhancer 1	12–19	UM+M	*TERT* prom * sex	wt vs C228T vs C250T	**0.033**	1.646	0.195
enhancer 1	17	UM	*TERT* rs2853669 * age	TT vs CT	**0.042**	4.372	0.058
		M	*TERT* rs2853669 * sex	TT vs CT	**0.039**	3.349	0.089
enhancer 2	mean 05–08	M	*TERT* rs2853669 * sex	TT vs CT	**0.037**	2.591	0.130
	05	M	*TERT* rs2853669 * sex	TT vs CT	**0.016**	3.606	0.078
	37	M	*TERT* rs2853669 * sex	TT vs CT	**0.017**	3.984	0.066
	38	M	*TERT* rs2853669 * age	TT vs CT	**0.017.**	4.478	0.053
			TERT rs2853669 * sex	TT vs CT		3.777	0.072
enhancer 4	07–08	UM	*TERT* rs2853669 * age	TT vs CT	**0.050**	3.840	0.061
	12	UM	*TERT* rs2853669 * age	TT vs CT	**0.050**	4.167	0.064
promoter	72–83	UM+M	Ki-67 * sex	≤ 50% vs > 50% Ki-67	**0.023**	0.178	0.673
enhancer 1	12–19	UM+M	Ki-67 * age	≤ 50% vs > 50% Ki-67	**0.029**	0.483	0.488

M: methylated, prom: promoter, UM: unmethylated
